# Optimizing fluorescent protein trios for 3-Way FRET imaging of protein interactions in living cells

**DOI:** 10.1038/srep10270

**Published:** 2015-07-01

**Authors:** Brandon L. Scott, Adam D. Hoppe

**Affiliations:** 1 Department of Chemistry and Biochemistry, South Dakota State University, Brookings, South Dakota 57007

## Abstract

Powerful new methods have extended FRET microscopy to the imaging of three or more interacting proteins inside living cells. Here, we compared widely available fluorescent proteins to find the best trio for 3-Way FRET imaging. We focused on readily available cyan, yellow, and red proteins that have high quantum yields, large extinction coefficients and good photostability, which defined these candidate proteins: CyPet/mTFP1/mTurqoise2, mCitrine/YPet, and TagRFP/TagRFPt/mRuby2/mCherry. By taking advantage of the high structural similarity across the fluorescent proteins, we generated structurally similar, but photophysically distinct donor/acceptor and triple fluorophore fusion proteins and measured their FRET efficiencies inside living cells. Surprisingly, their published photophysical parameters and calculated Förster distances did not predict the best combinations of FPs. Using cycloheximide to inhibit protein synthesis, we found that the different FP maturation rates had a strong effect on the FRET efficiency. This effect was pronounced when comparing rapidly maturing yellow and slowly maturing red FPs. We found that red FPs with inferior photophysics gave superior FRET efficiencies because of faster maturation rates. Based on combined metrics for the FRET efficiency, fluorophore photophysics and fluorophore maturation we determined that Turqoise2, YPet and Cherry were the best available FPs for live cell 3-Way FRET measurements.

Cellular signaling relies on organized molecular networks to relay information through protein binding events. New methods are being developed to access the dynamic and spatial organization of these networks^1^,^2^,^3^,^4^. Of these approaches, Fluorescence Resonance Energy Transfer (FRET) microscopy is a powerful tool to image the localization and dynamics of protein-protein interactions inside living cells^5^,^6^,^7^. Key examples include imaging experiments that demonstrate the activation of small G-proteins of the Ras, Rho, Arf, and Rab families, heterotrimeric G-proteins, and signaling receptors^8^,^9^,^10^,^11^,^12^. To reach its full potential for capturing network dynamics, FRET must be able to image multiple protein interactions within single living cells. Methods capable of imaging three interactions have been developed^13^,^14^,^15^ and recently a generalized theory/method for imaging FRET between two or more fluorophores, called N-Way FRET, has been developed^16^. Improved FRET imaging of molecular networks should be possible through the combination of these methods with optimized fluorescent protein tags.

The discovery and engineering of new fluorescent proteins (FPs) has created a large palette of possible molecules for N-Way FRET microscopy. Furthermore, many new FPs display outstanding brightness, owing to large extinction coefficients and high quantum yields, making them excellent candidates for FRET microscopy. However, quantitative N-Way FRET imaging imposes additional constraints on the selection of FPs (for a review, please see^17^,^18^). Specifically, the overlap of the donor’s (bluer fluorophore) emission spectrum with the acceptor’s (redder fluorophore) excitation spectrum determines the strength of FRET, (e.g the FRET efficiency) with respect to the molecular distance separating the donor and acceptor. Thus, the strength of a FRET signal depends on the choice of fluorophores. Previous work^13^,^15^,^16^ has demonstrated that the cyan, yellow and red FPs meet the criteria of spectral overlap for FRET at distances compatible with typical molecular complexes such as the EGFR/Grb2/Cbl interaction^13^, and the oligomerization of HIV-Gag^16^. These experiments illustrate that while the spectral overlap for the cyan-yellow pair is larger than for the cyan-red pair; the FRET efficiency for each possible interaction is large enough to be measured.

The photophysical parameters that determine the FRET efficiency for a given donor/acceptor pair are described by the Förster Equation, equation (1),


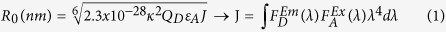


where J is the normalized overlap integral between the donor’s emission and acceptor’s excitation spectra, and the FRET efficiency is 0.5 at the Förster distance (R_0_). Fluorescent proteins in the same spectral class often have very similar excitation and emission spectra, but differ in brightness, a function of the extinction coefficient (ε) and quantum yield (Q) (Table 1). For acceptors with similar excitation spectra, the FP that exhibits a larger extinction coefficient is predicted to be a superior acceptor because of an increase in the overlap integral. For donors with similar emission spectra, the FP that exhibits a larger Q_D_ is predicted to be the better donor as seen in equation (1). The orientation between the fluorophores (κ^2^) has an impact on R_0_, but since it is unknown it is often assumed to be isotropic (κ^2^ = 2/3). Using other values of κ^2^ will change the magnitude of R_0_, but the trends between the different FP pairs will remain the same. In equation (2), the predicted FRET efficiency of different FP pairs can be compared if the distance separating the chromophores (r) is held constant.


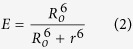


In addition to spectral overlap, parameters such as photostability and chromophore maturation can contribute significantly to the signal strength in FRET experiments; however these are not accounted for in equation (1), and need to be addressed experimentally.

In this work, we took advantage of the high structural similarity of the fluorescent proteins^19^,^20^ to create a system to optimize fluorophore selection for 3-Way FRET experiments. This system relied on expressing and imaging 2 and 3 fluorophore fusion proteins in living cells that should produce similar distances between the chromophoric centers, thereby allowing comparison of the biochemical and photophysical performance of FRET pairs and trios. We used acceptor photobleaching to quantify the FRET efficiency of each pair because it can be performed rapidly on many cells and color combinations without the need for instrument specific calibrations. This system of linked FP fusions also enabled us to quantify the relative maturation rates and address the contributions of donor and acceptor maturation on the FRET efficiency. We restricted our efforts to FPs from the Cyan (C), Yellow (Y) and Red (R) categories since these colors cover the most commonly developed FRET biosensors and FP fusions currently in use. These categories consisted of: C) CyPet/mTFP1/mTurqoise2 (Tq2), Y) mCitrine/YPet, and R) TagRFP/TagRFPt/ mRuby2/mCherry. Across these commonly used molecules, we found that significant improvements in multicolor FRET could be attained by overcoming relatively small deficiencies in photophysics, maturation rate and FRET couplings of these fluorophores.

## Experimental Methods

### Construction of the fluorescent fusion proteins

Fluorescent proteins (FP) used in this study included: the cyan fluorescent proteins (CFP) mTurquoise2 (Addgene plasmid 36201), mTFP1 (Allele Biotechnology) and CyPet (Addgene plasmid 22784), yellow fluorescent proteins (YFP) YPet (Addgene plasmid 22781) and mCitrine, red fluorescent proteins (RFP) mCherry, mRuby2 (Addgene plasmid 40260), TagRFP (Evrogen) and TagRFP-t. Donor only parent plasmids were generated by PCR amplification of the FP and AgeI/MfeI digestion into the Clontech-C1 plasmid. Structurally equivalent linked constructs were generated using the 8 amino acid linker TSLQEFGT between the donor and acceptor; the donor plasmid was cut with BgllI/MfeI and the PCR 5'-Linker-Acceptor product was cut with BamHI/MfeI. BglII removes the stop codon from the first FP and keeps the second FP in frame, and the BglII/BamHI sticky ends produce a new restriction site allowing the same enzymes to be used to add the third FP to produce the FRET trio. All constructs were sequenced and verified. All restriction enzymes were obtained from Promega, Madison, WI.

Primers used to make constructs

FP = mTq2, mTFP1, CyPet, YPet, mCit, mCherry

5'AgeI-FP:GATCCACCGGTCGCCACCATGGTGAGCAAGGGCGA

5'Linker Primers

5'FP: CACGGGATCCACTAGTCTGCAGGAATTCGGTACCATGGTGAGCAAGGGCGA

5'TagRFP: CACGGGATCCACTAGTCTGCAGGAATTCGGTACCATGGTGTCTAAGGGCGAAG

5'mRuby2: GCCTCCAATTGTTAAGATCTCTTGTACAGCTCGTCCATCCC

Reverse Primers: encodes bglll-TAA-mfel after FP

FP = mTq2, mTFP1, CyPet, mCit, mCherry

3'FP: GCCTCCAATTGTTAAGATCTCTTGTACAGCTCGTCCATGCC

3'YPet: GCCTCCAATTGTTAAGATCTCTTATAGAGCTCGTTCATGCCC

3'TagRFP: GCCTCCAATTGTTAAGATCTATTAAGTTTGTGCCCCAG

3'mRuby2: GCCTCCAATTGTTAAGATCTCTTGTACAGCTCGTCCATCCC

Primers to mutate the N-terminus of the red FPs:

5'Ruby_L, ATGGTGTCTAAGGGCGAAGAGGATAACATGGCCCTGATCAAGGAAAATATGCGT

5'TagR_L, ATGGTGTCTAAGGGCGAAGAGGATAACATGGCCCTGATTAAGGAGAACATGCAC

5'Che_S, ATGGTGAGCAAGGGCGAGGAGATCATCAAGGAGTTCATGCGC

### Cells and Transfection

COS-7 cells were obtained from the ATCC (Manassas, VA) and maintained at 37 °C under 5% CO_2_ in Dulbecco’s Modified Eagle Medium (HyClone, supplemented with 10% heat-inactivated CCS, 100 U/mL penicillin and 100 μg/mL streptomycin). Cells were plated at ~3 × 10^5^ cells onto 25 mm round No. 1.5 coverslips (Fisherbrand) and transfected with jetPEI transfection reagent (Polyplus Transfection, Strasbourg, France) according to the manufacturers recommendations. Media was replaced 16 hours after transfection and cells were imaged 24 hours after transfection. Cycloheximide (>94% TLC grade, Sigma-Aldrich, St. Louis, MO) was used in specified experiments to test the maturation of FPs, 100 μg/ml CHX was added to cells 16 hours after transfection, and cells were imaged after 5 hours.

### Imaging

A custom-built iMIC (FEI Munich GmbH) was used in this study and is described in detail elsewhere^16^. Briefly, the system consisted of an oligochrome module for excitation with the ability to quickly switch to collimated laser illumination for photobleaching steps. The system consisted of three emCCD cameras (2 – Andor iXon 885 and 1 - Andor iXonX3 885), allowing capture of fluorescence data from three fluorophores simultaneously.

### Preprocessing

All calculations were performed in Matlab (versions 7.3 and 2014a, Mathworks, Natick, MA) in conjunction with the DipImage toolbox (version 2.5.1 http://www.diplib.org/, Quantitative Imaging Group, Delft University of Technology, Netherlands). All data images were preprocessed by subtracting camera bias and shade-correcting the images as previously described^21^. Briefly, images for each camera were captured while blocking all light to obtain the bias level. Residual background was subtracted from cell-free regions, if present (generally less than 5% of the cellular signal). For shade correction, images were captured of a thin solution of a fluorescent protein mixture. The measured illumination pattern across the field of view for each excitation was used to normalize bias-corrected raw data. Images were registered as described previously^22^. In brief, fluorescent fiducial markers (Green PS-Speck beads, Life Technologies, Grand Island, NY) immobilized on glass were simultaneously imaged on each detector to create a grid pattern to sample the field of view. A polynomial transformation vector was determined to register images, generally aligning to the yellow channel.

### FRET efficiency measurements by acceptor photobleaching

**Figure 1:**
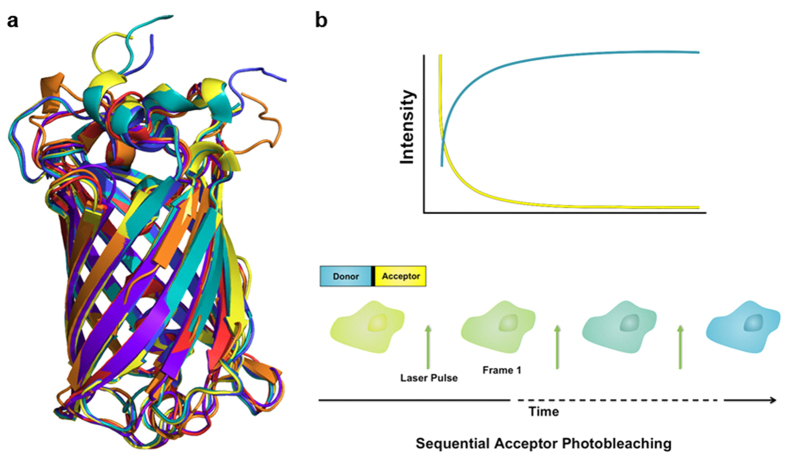
Fluorescent protein structural homology and general experimental approach. **a**. The pdb files for mCherry (red), TagRFP (orange) Citrine (yellow), CyPet (cyan), mTFP1 (purple), and Tq2 (blue) were aligned to mCherry and displayed in PyMOL, the RMSD values range from 0.49 – 1.09 Å. The high sequence and tertiary structure homology of the FPs provides evidence that the chromophores will be at a very similar distance in all constructs with a common linker allowing for direct comparison based on the experimental FRET efficiencies. **b.** The FRET efficiency is determined for a linked construct following acceptor photobleaching. As the acceptor is bleached, the donor is dequenched, and the change in donor fluorescence is used to calculate the FRET efficiency. Images are also collected in the acceptor channel to ensure complete photobleaching occurs.

Determination of the FRET efficiency for the linked constructs was achieved by selectively photobleaching the acceptor, either RFP or YFP using a 561 nm or 515 nm laser (FEI Munich GmbH) (and diagrammed in Fig. 1b). Both lasers produced collimated illumination fields in the sample plane via a lens that focuses the beam onto the back focal plane of the objective lens. FRET efficiency was computed as the increase in CFP or YFP fluorescence following bleaching of RFP and the increase in CFP fluorescence following bleaching of YFP. The intensity of the donor and acceptor were recorded during the gradual photobleaching of the acceptor. Regions of Interest (ROI) were chosen on the preprocessed data, and an intensity-based binary mask tightly outlined the cellular volume. The quantified data represents the integrated intensity normalized over the area of the cell to eliminate error associated with different sized regions. The bleaching illumination and duration were optimized for each FP pair to ensure complete bleaching of the acceptor and incidental photobleaching of the donor was less than 5% (see Supplemental Fig. S1 online). The same conditions were used for all acceptors of the same spectral class (e.g. all constructs with red acceptor were bleached with the same conditions). This paradigm permitted quantification of the characteristic FRET efficiency for each construct tested and is translatable to any imaging system.

### Photostability of cyan FPs

Cells expressing one of the cyan FPs were imaged using similar illumination intensity as in the acceptor photobleaching experiments for 20 frames at 8 fps. The average intensity of the cell was quantified for each time point.

### Structural Homology

PDB files were obtained for mCherry (2H5Q), TagRFPt (3T6H), Citrine (3DPZ), CyPet (3GEX), mTFP1 (2HQK) and mTq2 (3ZTF). All structures were aligned to mCherry in PyMOL using the align command.

### Statistics

Statistical significance (p < 0.05) was judged by One-way or Two-way ANOVA when appropriate, followed by Tukey HSD post hoc comparison of means using GraphPad Prism version 6.0 e for Mac, GraphPad Software, San Diego California USA, www.graphpad.com.

## Results and Discussion

To compare the performance of candidate donors and acceptors, we took advantage of the high degree of structural similarity shared amongst all FPs^19^ to generate structurally similar multiple FP fusion proteins. The degree of structural similarity of the FP subunits used in making these proteins can be seen in a backbone overlay of FPs with reported crystal structures which all aligned with RMSD < 1.09 Å (Fig. 1a). We then generated series proteins consisting of double and triple FP subunits positioning the chromophore centers at similar distances via an 8 amino acid linker. During this process, we noted that there was a 4 amino acid stretch at the N-terminus of Cherry that was not present in the other red constructs. We accounted for this by adding or removing this stretch to make equivalent linkers (termed long and short, Fig. 3a). To determine the characteristic FRET efficiency for each construct we expressed the double or triple linked constructs in cells and photobleached the yellow (or red) acceptors with a laser while measuring the donor intensity (Fig. 1b). The FRET efficiency (E) was calculated using equation (3), where F_D_ is the donor intensity before (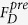) and after photobleaching (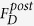).


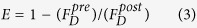


We chose this method because it can be performed rapidly on many individual cells without instrument specific calibration steps. One potential pitfall of acceptor photobleaching is the incidental photobleaching of the donor. However, control experiments demonstrated this was small (generally <5%, see Supplemental Fig. S1 online) and we chose not to make corrections to the data since FRET can modulate the photobleaching rate of both the donor and the acceptor, thereby complicating the correction and introducing errors^18^.

### Analysis of Cyan-Yellow FRET Pairs

**Figure 2:**
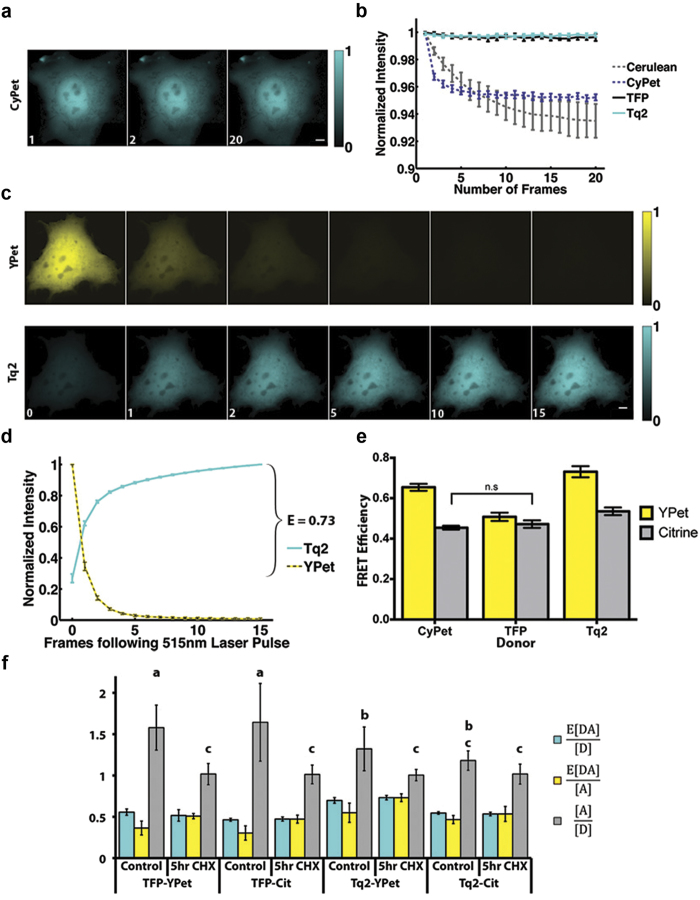
YPet is the optimal yellow FRET acceptor for cyan donors. **a**. Cyan FPs have differential photostabilities under nominal excitation. COS-7 cells were transfected with cytosolic CyPet and continuously imaged for 20 frames (100 ms per acquisition) with 442 nm excitation (125 μW illumination). All scale bars display 5 μm. **b**. Normalized intensity traces for ROI around individual cells expressing different cyan donors. Notice that CyPet exhibits two photostable states, which is problematic for imaging cellular dynamics on the timescale of seconds to minutes. Error bars report standard deviation with N = 20. **c.** COS-7 cells were transfected with Tq2-YPet and the acceptor was selectively photobleached by collimated 515 nm laser illumination (1,500 ms pulse at 72 W/cm^2^; 15 cycles). **d.** The average intensity for ROIs around individual cells over the time course of the acquisition. FRET efficiency is calculated as the change in donor intensity before and after the acceptor is photobleached, resulting in a FRET efficiency of 0.73 ± 0.02. **e.** Summary of characteristic FRET efficiencies for various cyan/yellow pairs showing selection of YPet as the best yellow acceptor. All error bars are standard deviation with N = 30. All bars are statistically significant (p < 0.05), using Two-way ANOVA followed by Tukey HSD comparison of means, except where noted. **f.** COS-7 cells expressing various cyan/yellow pairs were treated with 100 μg/mL cycloheximide (CHX) for 5 hrs in order to block synthesis of new protein. Linked molecules are assumed to be expressed at equal ratios; however, because of the differential maturation rates of the FPs this may not the case. Indeed, prior to treatment with CHX Tq2-YPet has a [Y] to [C] ratio of 1.32 and TFP-YPet is 1.58. Following treatment with CHX for 5 hours, the [Y] to [C] ratio is 1 to 1 for all constructs. All error bars are standard deviation with N = 10. Ratios before and after CHX treatment not connected by the same letter are statistically significant (p < 0.05), using Two-way ANOVA followed by Tukey HSD comparison of means. (Significance for E[DA]/[A] and E[DA]/[D] are not shown for clarity.)

We began by comparing the most commonly used cyan and yellow FRET pairs. Prior to measuring the FRET efficiencies for these constructs, we were concerned that the previously documented transient photoconversion of Cerulean and CFP derivatives could limit their utility as donors for dynamic FRET measurements^23^,^24^. Indeed, COS-7 cells expressing cyan FPs revealed a photoconversion to an apparently dark state under ambient imaging conditions for CyPet and Cerulean (as previously reported^25^), but not TFP (as previously reported^26^) or Tq2 (Fig. 2a,2b). We estimate that this effect could result in an error in FRET efficiency approaching 10% (compare Fig. 2b,2d). This phenomenon would be problematic during dynamic imaging, as the apparent FRET efficiency would be fluctuating as a result of donor photoconversion independent of the biochemistry that is being monitored. Furthermore, the strength of photoconversion depends on the illumination intensity and the photoconversion is reversible^23^,^25^,^27^. While these effects are relatively small for typical widefield imaging conditions, they do pose a limitation on the achievable precision of FRET microscopy and could have significant effects in confocal arrangements. A cyan donor that can achieve a similar FRET efficiency without this photoconversion phenomenon would improve the reliability of dynamic FRET microscopy.

A second concern was the photoconversion of YFPs. It has been reported that yellow FPs, especially eYFP, may photoconvert to a CFP-like state upon bleaching with 515 nm illumination thereby creating artifacts in the measured FRET efficiency. However, this phenomenon is greatly reduced in newer variants such as Venus or Citrine^28^. In Supplementary Fig. S2 online, we quantified the intensity of YPet in the CFP and RFP channels following 561nm and 515nm illumination, and report no measurable photoconversion of YPet.

To compare FRET efficiencies of the cyan-yellow pairs, cells expressing a cyan-yellow (e.g. Tq2-YPet) linked construct were subjected to progressive acceptor bleaching using 15 pulses of 515 nm illumination, (72 W/cm^2^, 1,500 ms duration, Fig. 2c,2d). For the Tq2-YPet example, the increase in cyan fluorescence intensity resulted in a measured FRET efficiency of 0.73 ± 0.03 per equation (3) (Fig. 2d). Repeating this measurement for all C-Y pairs showed that YPet gave FRET efficiencies that were 33% larger than Citrine when the donor was Tq2, and 44% larger with CyPet as the donor (Fig. 2e and see Supplementary Fig. S3 online). These results are generally consistent with the larger R_0_ of YPet over Citrine, owing to YPet’s larger extinction coefficient (Table 1 and 2). For example, the 50-70% FRET efficiencies measured for these constructs suggests inter-chromophore distances of ~4-5 nm. Using equation (2) and the data in Table 2, we would predict a 7-15% increase in the FRET efficiencies for YPet over Citrine (assuming 5 nm separation distance). While this expected difference was observed when TFP was the donor (9% predicted, 7% measured enhancement for YPet over Citrine, Fig. 2e), the FRET efficiency difference with CyPet or Tq2 as donors was much larger than expected. This result indicates that Citrine and YPet likely have physical interactions with Tq2 or CyPet that change either the orientation or the distance between chromophores, and that these interactions do not occur when TFP is the donor.

We examined the potential increase in FRET efficiency with either CyPet or Tq2. Again assuming a separation distance of 5 nm for both pairs, we would predict a 20% increase in the FRET efficiency with either acceptor by replacing CyPet with Tq2. In the Citrine constructs we measured a 15% increase, but for YPet we measured a 10% increase (Fig. 2e). Together, these data indicate that much of the enhancement is likely due to an affinity between YPet and CyPet that occurs less with Tq2 but not with Citrine, as previously reported for YPet and CFP derivatives^29^,^30^,^31^.

Alternatively, differential maturation rates of the donor and acceptor FPs could create a similar effect. For example, these differences could be explained if Citrine maturation was slower than YPet but similar to TFP (and Tq2 and CyPet were fast). To test the possibility that that differences in donor and acceptor maturation rate modulated the measured FRET efficiencies, we used N-Way FRET to measure the molar ratio between donor (D) and acceptor (A) as well as the apparent FRET efficiencies (e.g. E[DA]/[A] and E[DA]/[D])^21^. By treating cells with cycloheximide (CHX) to inhibit protein synthesis, we provided both FPs an opportunity to reach their fully matured states (Fig. 2f). In all cases, CHX treatment increased the apparent acceptor FRET efficiency, E[DA]/[A] and decreased the molar ratio [A]/[D], indicating that the maturation of YPet and Citrine was faster than either TFP or Tq2 and that when synthesis was arrested, they could both fully mature (Fig. 2f). Since the cyan fluorophores were rate limiting, no changes in E[DA]/[D] were observed indicating that the differences observed in the photobleaching experiment could not be explained by maturation differences between Citrine and YPet. Furthermore, consistent with the photobleaching data, YPet showed stronger FRET as measured by E[DA]/[D] (a measure equivalent to photobleaching) and E[DA]/[A] than Citrine for Tq2 as the donor, but the differences were much smaller when TFP was the donor. Together, these data confirm that there is a physically distinct arrangement of chromophores for Tq2-YPet that is not present in TFP-YPet, TFP-Cit or Tq2-Cit.

Overall, the superior photostability, maturation rate and measured FRET efficiencies suggest that Tq2 is currently the best donor for the C-Y pair. Additionally, the comparable photostabilities of YPet and Citrine^32^, and the higher FRET efficiencies of the former, suggest that YPet is the better acceptor for the C-Y pair.

### Analysis of Yellow-Red FRET Pairs

**Figure 3:**
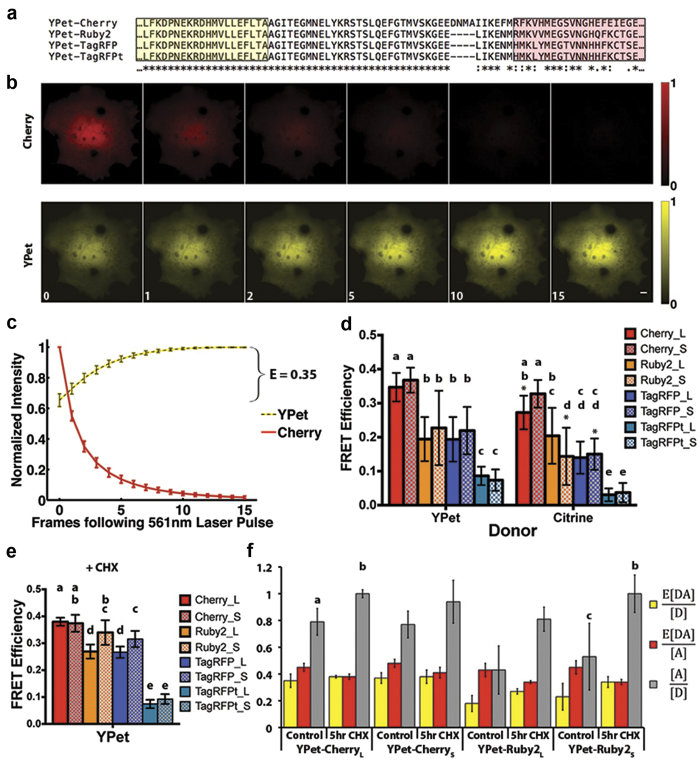
Cherry is the optimal red FRET acceptor for yellow donors. **a.** The sequences of YPet-red constructs were aligned using Clustal Omega, shown here around the linker region, with beta barrel residues of the FPs highlighted. The remaining residues have a predicted coiled secondary structure. Notice the additional residues (8-11 DNMA) in mCherry compared to the other red proteins. In order to make a robust comparison of FRET efficiencies, constructs denoted as YPet-Red_L_ or Cit-Red_L_ have DNMA inserted in Ruby2, TagRFP and TagRFPt. In YPet-Red_S_ or Cit-Red_S_ DNMA has been deleted in mCherry. **b**. In COS-7 cells expressing YPet-Cherry_L_ (WT), the acceptor was selectively photobleached by collimated 561 nm laser illumination (4,500 ms pulse at 95 W/cm^2^; 15 cycles). **c**. The average intensity for ROIs during the acquisition resulting in a FRET efficiency of 0.35 ± 0.06. **d**. Characteristic FRET efficiencies for various yellow/red pairs, all error bars are standard deviation with N ≥ 10. YPet-Red bars not connected by the same letter are significantly different (Note: No comparison is made across donor at this point) (p < 0.05) using One-Way ANOVA followed by Tukey HSD post hoc comparison of means. Asterisk indicates significant difference between YPet and corresponding Citrine construct (p < 0.05) using Two-Way ANOVA followed by Tukey HSD post hoc comparison of means. **e.** Characteristic FRET efficiencies measured by acceptor photobleaching for YPet-Red constructs following treatment with 100 μg/mL CHX for 5 hrs. **f.** N-Way FRET analysis of various YPet-Red constructs following CHX treatment demonstrating the differential maturation rates of the FPs. mCherry-YPet has a higher control ratio of 0.8 compared to mRuby2-YPet, which is 0.4. Following treatment with CHX for 5 hours, the [R] to [Y] ratio is 1 to 1 for all constructs except YPet-Ruby2_L_ which increases to 0.8 (p < 0.05) Two-way ANOVA followed by Tukey HSD post hoc comparison of means. (Significance only shown for molar ratios of wildtype constructs for clarity.)

Given their similar photophysical parameters (Table 1), but distinct differences in FRET efficiencies that we observed when pairing with Tq2, we chose to compare both YPet and Citrine as donors for FRET pairs containing red FP acceptors. During assembly of the Y-R linked constructs, we noticed a four amino acid addition in the flexible region of Cherry’s amino terminus that was not present in the other red FPs (Fig. 3a). To make a robust comparison with minimal sequence variation, we created constructs with the deletion of amino acids 8-11 (∆DNMA) in Cherry resulting in the short constructs (denoted by subscript S) and we added the same amino acids (+DNMA) to Ruby2, TagRFP and TagRFPt resulting in long constructs (denoted by subscript L). This strategy also allowed us to control for any unexpected contributions of the linker region to changing chromophore distances, orientations or maturation rates.

The FRET efficiencies of the resulting constructs were determined using acceptor photobleaching with 15 pulses of 561 nm illumination (95 W/cm^2^, 4,500 ms duration, Fig. 3c and see Supplementary Fig. S4 online). In these experiments, we observed three surprising but striking phenomena. First, as a class, the Yellow-Red FRET pairs have the largest Förster distances (Table 2), but all the Cyan-Yellow pairs have measured FRET efficiencies that are much higher, (compare Fig. 2e and Fig. 3d). Second, despite both YPet and Citrine having nearly identical Förster distances for nearly every Y-R FRET pair measured (Table 2), YPet gave higher FRET efficiencies than Citrine (Fig. 3d). These data indicate that YPet was a better yellow FP for Y-R FRET, possibly because of enhanced dimerization between YPet and other FPs, consistent with the observed trends of the Cyan-Yellow pairs.

Third, and perhaps most striking, was the fact that Ruby2 and TagRFP gave lower FRET efficiencies than Cherry. Despite our concerns about the 4 amino acid insertion on the amino terminus of mCherry, no statistically significant differences in FRET efficiency were observed between the long and short constructs with YPet as the donor (Fig. 3d), as a result we report the FRET efficiency of the wildtype constructs. Specifically, YPet-Cherry exhibited the highest FRET efficiency of 0.35 ± 0.04, which was greater (p < 0.05) than YPet-Ruby2 (0.23 ± 0.11) and YPet-TagRFP (0.22 ± 0.07) (Fig. 3d). Based on published parameters, Cherry’s Förster distance was predicted to be shorter than Ruby2 and TagRFP, but the same as TagFRPt (Table 1 and 2). Based on the calculated Förster distances (Table 2), YPet-Ruby2 and YPet-TagRFP should have produced ~33% higher FRET efficiency than YPet-Cherry (assuming a chromophore separation distance of 6.5 nm); however, YPet-Ruby2 and YPet-TagRFP FRET efficiencies were lower than YPet-Cherry by ~50% and had large cell-to-cell variability (Fig. 3D). Furthermore, YPet-TagRFPt FRET efficiencies were even lower (only about ~10-25% of that obtained with Cherry). We speculated that Ruby2 and the TagRFPs were maturing much more slowly than YPet and this might have caused these differences. To test this idea, cells expressing the linked constructs were treated with cycloheximide (CHX) for 5 hours to block new protein synthesis and to allow translated FPs to mature. Using acceptor photobleaching to determine the FRET efficiency (Fig. 3e) we found that CHX treatment increased the FRET efficiency of both forms of the YPet-Ruby2 and YPet-TagRFP constructs while decreasing their cell-to-cell variability, but had no effect on the YPet-Cherry constructs (compare Fig. 3d,3e). These data are consistent with Cherry maturing at a similar rate to YPet, and faster than Ruby2 or the TagRFPs. Similar to our approach for the C-Y pairs, we applied N-Way FRET analysis^16^ to quantify the ratio of acceptor to donor as well as the apparent FRET efficiencies (e.g. E[DA]/[A] and E[DA]/[D])^21^. Here, we observed the same phenomena as observed with C-Y pairs, but now reversed across the N-Way FRET parameters reflecting the fact that the faster maturing molecule was now the donor. Specifically, upon CHX treatment we observed an increase in the E[DA]/[D] and [A]/[D] parameters and E[DA]/[A] remained unchanged (Fig. 3f). Although unexpected, our data suggest that Cherry, with its faster maturation rate and improved FRET efficiency is the best red FP for Y-R FRET.

### Analysis of Cyan-Red FRET pairs

**Figure 4:**
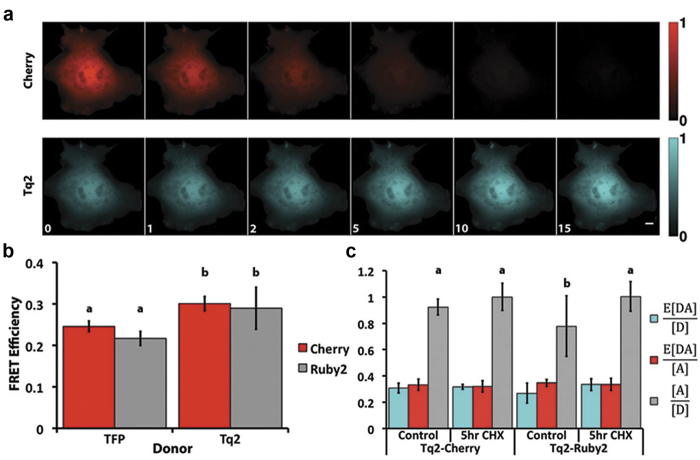
Tq2 is the optimal cyan FRET donor and mCherry, which matures rapidly, is the optimal red acceptor. **a**. COS-7 cells were transfected with Tq2-Cherry, and the acceptor was selectively photobleached by collimated 561 nm laser illumination (4500 ms pulse at 95 W/cm^2^; 15 cycles). **b**. Summary of the average FRET efficiency for each cyan-red construct. FRET efficiency was measured using acceptor photobleaching for either TFP or Tq2 as the donor and mCherry or Ruby2 as the acceptor. Error bars are standard deviation with N = 30. Bars not connected by the same letter are statistically significant (p < 0.05) Two-way ANOVA followed by Tukey HSD post hoc comparison of means. **c**. The red fluorophore maturation is compared following treatment with 100 μg/mL CHX for 5 hrs. The [R] to [C] ratio recovered from N-way FRET increases from 0.78 to approximately 1 at steady state for Tq2-Ruby2, and increases from 0.92 to 1 for Tq2-Cherry. Notice E[DA]/[D] increases with cycloheximide treatment as more acceptors mature, but E[DA]/[A] is unchanged; error bar N = 10. (p < 0.05) Two-way ANOVA followed by Tukey HSD post hoc comparison of means. (Significance for E[DA]/[A] and E[DA]/[D] not shown for clarity.)

To determine the best C-R FRET pair, we limited our measurements to the top two candidates from each color (Tq2/TFP and Cherry/Ruby2). As seen in Table 2, Tq2 and TFP have nearly identical R_0_ values when paired with red acceptors owing to the larger quantum yield of Tq2 offsetting the larger overlap integral of TFP. Despite having nearly identical predicted R_0_ values, Tq2 produced higher FRET efficiencies than TFP (Fig. 4b and see Supplementary Fig. S3 online). This result recapitulates the observation for C-Y FRET pairs where, Tq2 outperformed TFP (Fig. 2) despite having a shorter predicted R_0_ (Table 2); however, the increase in FRET efficiency of the C-R pair is much less than the C-Y pairs. This is further evidence of a structural difference between the Tq2-YPet construct that is not present when TFP is used or if YPet is replaced with a red FP. The maturation differences between Cherry and Ruby2 were reproduced in constructs with Tq2 (Fig. 4c). The concentration ratio increased from 0.76 to 1 for Tq2-Ruby2 and increased from 0.92 to 1 for Tq2-Cherry. Cherry remains the superior acceptor within living cells given its improved maturation rate, greater FRET efficiency with yellow donors, and its equivalent FRET efficiency with cyan donors. Thus, the theoretical predictions from published photophysical constants are a good place to start in optimizing, but given the current data it is necessary to test the constructs in living cells to characterize the fluorophores.

### Analysis of FRET Trios

**Figure 5:**
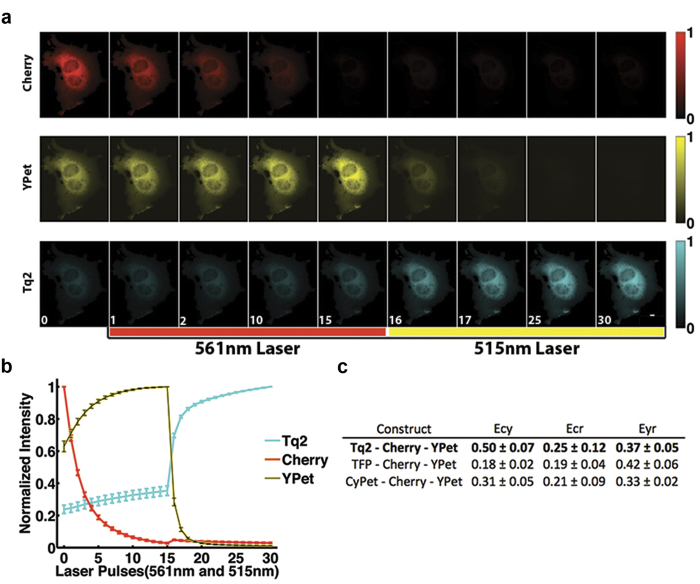
Tq2-Cherry-YPet is the optimal FRET trio. COS-7 cells were transfected with the best cyan, yellow and red FP linked trios. **a.** Here showing: Tq2-Cherry-YPet. The two acceptors were selectively and sequentially photobleached. Cherry was photobleached by collimated 561 nm laser illumination (4500 ms pulse at 95 W/cm^2^; 15 cycles), followed by YPet photobleaching by collimated 515 nm laser illumination (1500 ms pulse at 72 W/cm^2^; 15 cycles). **b**. The average intensity for ROIs during the acquisition shows the expected trends as the yellow and cyan donors are dequenched. **c**. Characteristic FRET efficiencies for various Cyan-Cherry-YPet constructs, all error bars are standard deviation with N = 30.

Based on the pairwise acceptor photobleaching results and maturation experiments, we predicted that Tq2, YPet and Cherry would be the optimal FPs for three-color FRET imaging in living cells. From the C-Y determination, we predicted that the largest changes in FRET efficiency would occur by varying the cyan FP. We tested this prediction by generating multiple triple FRET constructs with the arrangement: C-R-Y and then measured the three possible FRET efficiencies (C-Y, C-R, Y-R) using sequential photobleaching of the red and yellow FPs. The FRET efficiencies were measured following 5 hr CHX treatment to reduce the effects of differential FP maturation rates. These effects were minimal because the optimal FPs had similar maturation rates (Fig. 2,3,4). We first quantified the FRET efficiencies of the Tq2-Cherry-YPet construct by photobleaching Cherry and measuring the corresponding increases in both YPet and Tq2 fluorescence, which resulted in the Tq2/Cherry (0.25 ± 0.12) and YPet/Cherry (0.37 ± 0.05) FRET efficiency, respectively (Fig. 5b). The Tq2/YPet efficiency (0.50 ± 0.07) was determined by photobleaching YPet and measuring the change in Tq2 fluorescence (Fig. 5b). Replacing Tq2 with either TFP or CyPet resulted in a large decrease in the C-Y FRET efficiency confirming that Tq2, YPet and Cherry are the optimal trio. The interactions between Tq2 and YPet that result in a higher than predicted FRET efficiency do not influence the FRET efficiency of YPet/Cherry, because each trio has a similar Y/R FRET efficiency that are the same as the YPet-Cherry pairwise measurement.

## Conclusion

We have characterized a cohort of widely available fluorescent proteins for 3-color live cell FRET imaging. In this study we have found that photophysical parameters may not predict the optimal FRET trio. Rather, we found that differences in FP maturation rate, reversible photobleaching and structural interactions between FPs can affect the strength and reliability of the FRET efficiency. Together, these parameters define guiding principles for the selection of 3-Way FRET fluorophores for intermolecular and intramolecular FRET sensors.

We conclude that Tq2 is the best cyan FP; it demonstrated superior photostability and FRET efficiencies when compared against the other cyan FPs. Surprisingly, the large FRET efficiencies observed with Tq2 were likely due to surface interactions that reduce the donor and acceptor chromophore separation, and this was particularly strong when YPet was used. Previously, weak interactions that enhance dimerization have been reported as ways to increase the FRET efficiency of a given pair of FPs^11^,^33^. Our results for the Cyan-YPet FRET pair are consistent with structural interactions resulting in higher than predicted FRET efficiencies. TFP was predicted to have the highest FRET efficiency with the yellow FPs based on spectral overlap; however, both Tq2 and CyPet significantly outperformed it. Additionally, YPet gave FRET efficiencies much greater than we would predict over Citrine when paired with Tq2 and CyPet. Surprisingly this effect was not seen with TFP. The sequences of Tq2, CyPet, Citrine and YPet are all very similar to one another (92-95% identity), but TFP’s amino acid sequence diverges (33% identity) indicating that Tq2 and CyPet likely have similar surfaces that enhance dimerization with YPet leading increased FRET efficiency^30^,^31^,^33^. This result lends credence to the idea of boosting FRET by engineering weak interfaces that bring FPs close together, although caution must be used to not to create artifacts.

The surprising finding that Cherry was the best red FP highlighted the importance of having FRET partners with matching (and fast) maturation rates. Differences in maturation will affect both intra- and intermolecular FRET experiments by modulating the acceptor/donor ratio and therefore the apparent FRET efficiency. The donor:acceptor ratio is assumed 1:1 in intramolecular biosensors, but this is not achieved when there are large differences in maturation rates. For intermolecular interactions the distribution of free donors will be changing as acceptors mature, thereby artificially changing the apparent FRET efficiency. This effect is particularly confounding given that its strength will depend on the rate of FP synthesis and turnover within the cell and may be different depending on cell type. Rapidly induced FP fusions with large discrepancies in maturation rate (YPet relative to TagRFP) would be expected to produce the largest errors. The differences in maturation rates of Ruby2 and the TagRFPs may improve the range of protein turnover kinetics that can be measured in FP-timer experiments^34^, but they are not ideal for FRET experiments.

These results emphasize the importance of experimental determination of the optimized FPs. The published photophysical data and theoretical predictions alone do not address the photostability, maturation, or surface interactions of the FPs that alter the FRET efficiency. The development of new spectral variants such as mNeonGreen will likely lead to a wider color palette of FRET-suitable FPs for multi-color FRET experiments^35^. Our work illustrates that the construction and evaluation of linked molecules within cells by multiple methods provides a useful approach for benchmarking their utility for multi-color FRET microscopy.

## Additional Information

**How to cite this article**: Scott, B. L. and Hoppe, A. D. Optimizing fluorescent protein trios for 3-Way FRET imaging of protein interactions in living cells. *Sci. Rep.* **5**, 10270; doi: 10.1038/srep10270 (2015).

## Supplementary Material

Supplementary Information

## Figures and Tables

**Table 1:** Photophysical parameters of FPs that affect the FRET efficiency and photostability.

	Ex λ(nm)	Em λ(nm)	ε(mM^−1^cm^−1^)	Q	Photostability (s)	Ref
mCerulean	433	475	43	0.62	36	36
mTurqoise2	434	474	30	0.93	90	37
CyPet	435	477	35	0.51	59	29
mTFP1	462	488	64	0.85	110	38
mCitrine	516	530	77	0.76	49	39
YPet	517	530	104	0.77	49	29
TagRFP	555	584	100	0.48	37	40
TagRFP-t	555	584	81	0.41	337	41
mRuby2	559	600	113	0.38	123	42
mCherry	587	610	72	0.22	96	43

**Table 2:**
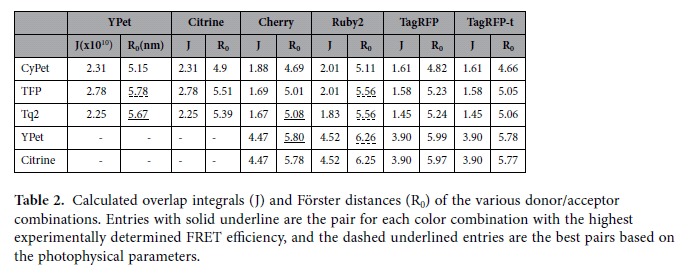
Calculated overlap integrals (J) and Förster distances (R_0_) of the various donor/acceptor combinations.
