# Supplementary material for: Optimizing fluorescent protein trios for 3-Way FRET imaging of protein interactions in living cells
Source: Sci Rep. 2015 Jul 1;5:10270. doi: 10.1038/srep10270 (PMC4487001; doi:10.1038/srep10270)
Supplement: Supplementary Information [file srep10270-s1.pdf]

Supplemental Info: Optimizing the fluorescent protein trio for 3-Way FRET imaging of protein interactions in living cells

Brandon L. Scott and Adam D. Hoppe*

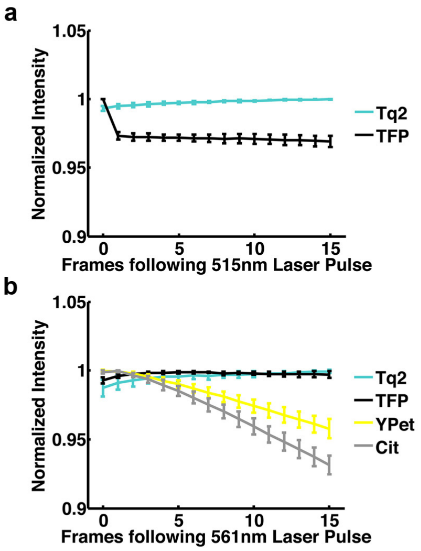

Figure S1. Negligible incidental donor bleaching occurs during the acceptor photobleaching sequence. Control experiments performed on COS-7 cells that express donor only FPs. **a.** COS-7 cells expressing Cyan (Tq2 and TFP) FPs were photobleached by collimated 515nm laser illumination (1,500 ms pulse at 72 W/cm^2; 15 cycles). Incidental photobleaching was less than 5% over the course of the experiment, and no photobleaching correction was required. **b.** COS-7 cells expressing Cyan (Tq2 and TFP) and Yellow (YPet and Citrine) FPs were photobleached by collimated 561nm laser illumination (4,500 ms pulse at 95 W/cm^2; 15 cycles). Incidental photobleaching was less than 5% over the course of the experiment with the exception of Citrine (7%), and no photobleaching correction was required. All error bars are standard deviation with N= 10.

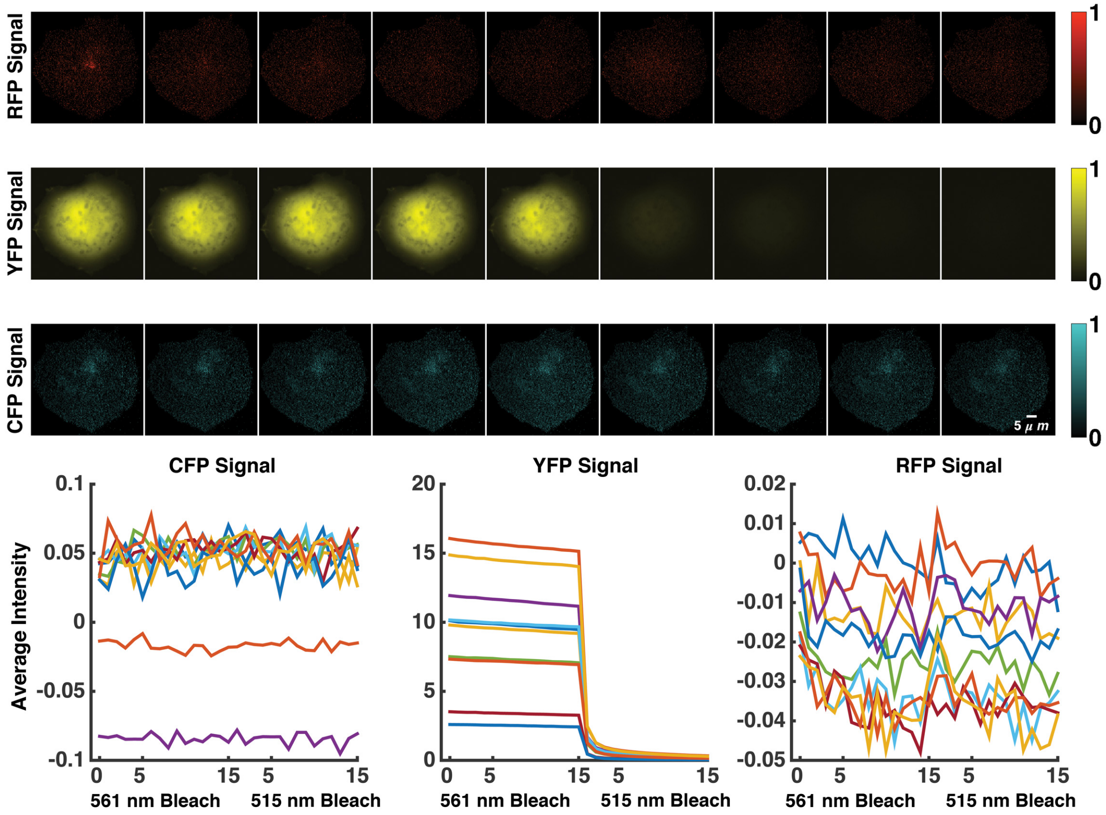

Figure S2. A CFP-like molecule is not detected during acceptor photobleaching sequence of YPet. Control experiments performed on COS-7 cells that express only YPet were photobleached by collimated 561nm laser illumination (4,500 ms pulse at 95 W/cm^2; 15 cycles) followed collimated 515nm laser illumination (1,500 ms pulse at 72 W/cm^2; 15 cycles). **b.** Average intensity of 10 cellular ROIs indicates that there is no increase in the CFP channel following bleaching with either the 561 or 515 nm lasers.

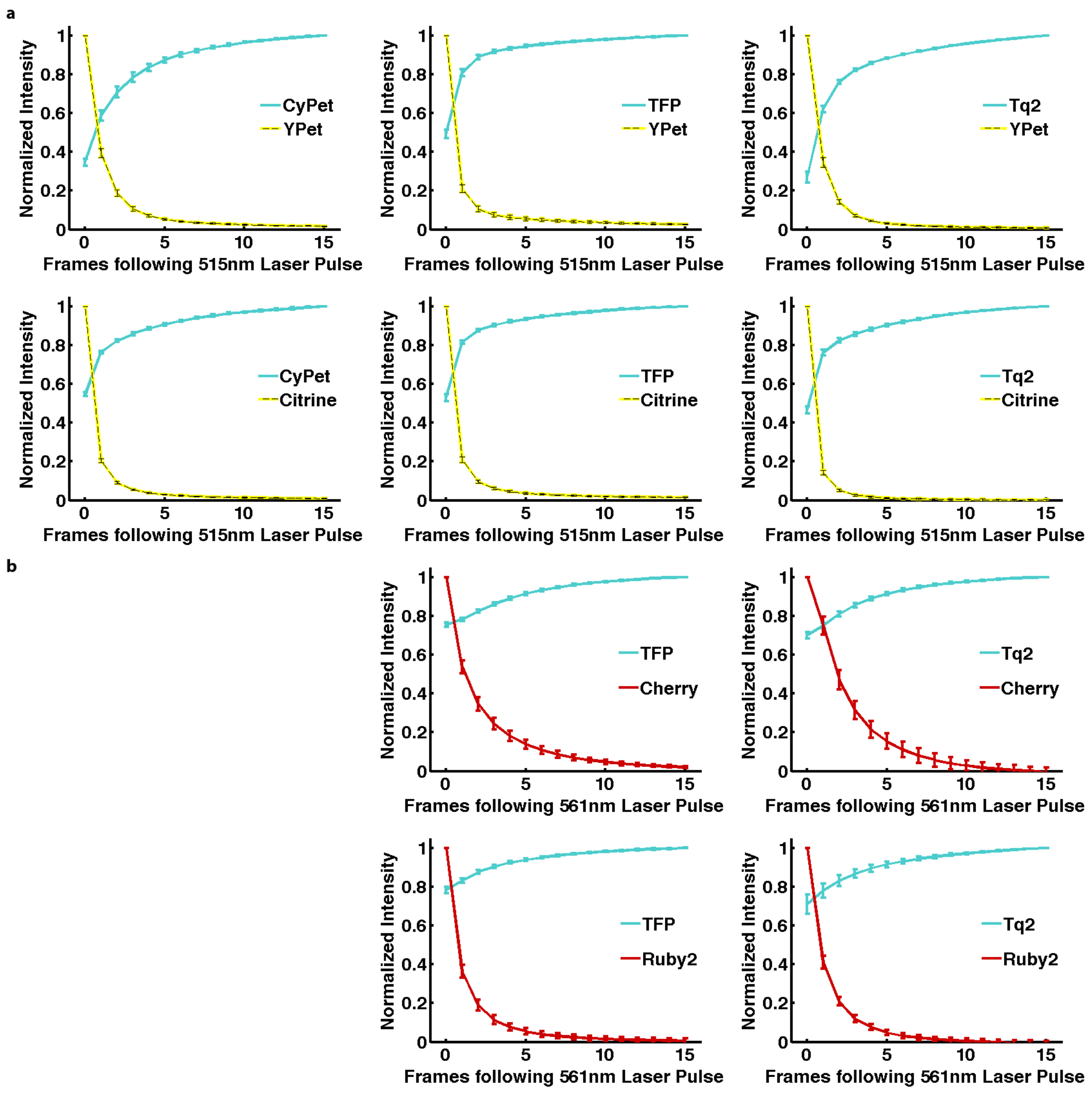

Figure S3. Quantified bleaching with cyan donors. a. Bleaching curves for data associated with Figure 2. **b.** Bleaching curves associated with Figure 4.

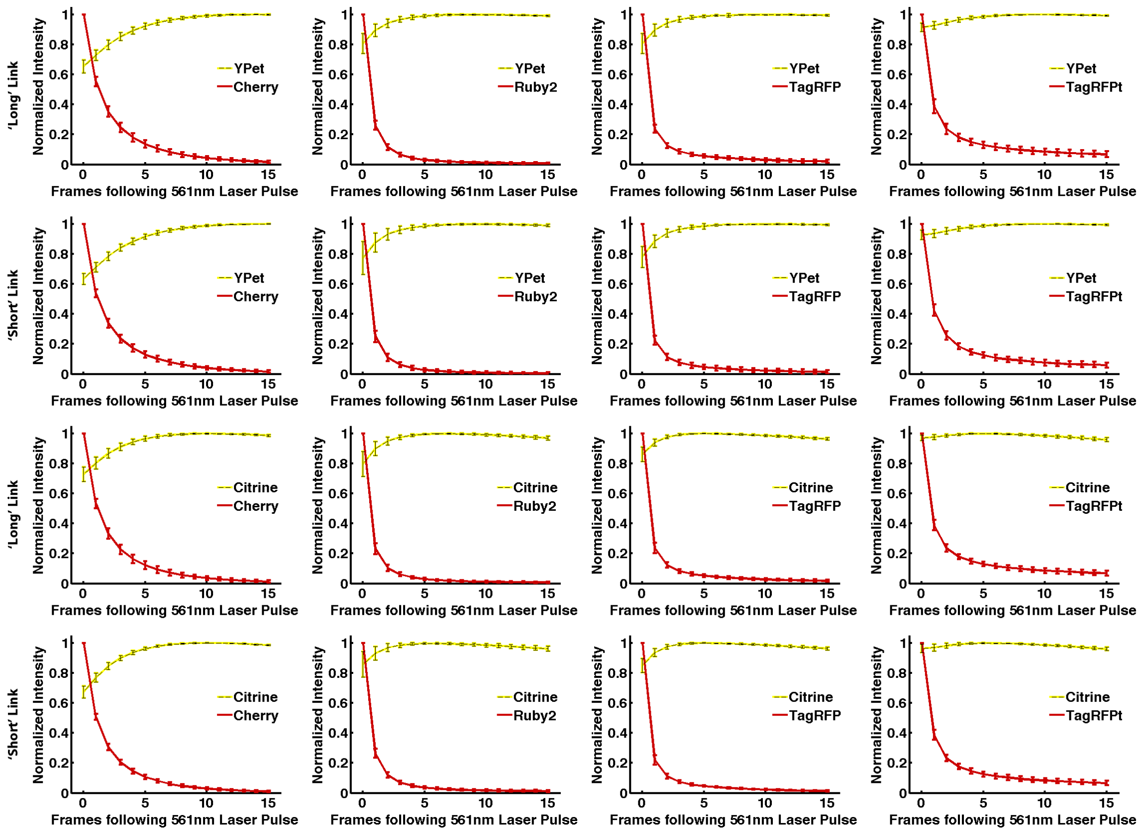

Figure S4. Quantified bleaching with yellow donors. Bleaching curves for the yellow-red constructs associated with Figure 3.
